# Paracentral Acute Middle Maculopathy and Central Retinal Venous Occlusion following Electrical Injury

**DOI:** 10.1155/2022/3699667

**Published:** 2022-04-16

**Authors:** Sahel Khazaei, Mehrdad Motamed Shariati, Naser Shoeibi, Mohammad Arjmand, Seyedeh Maryam Hosseini

**Affiliations:** ^1^Eye Research Center, Mashhad University of Medical Sciences, Mashhad, Iran; ^2^Sabzevar University of Medical Sciences, Heshmatie Hospital, Sabzevar, Iran

## Abstract

**Purpose:**

To report a case of central retinal vein occlusion (CRVO) and paracentral acute middle maculopathy (PAMM) following electric shock injury. *Case Description*. A 45-year-old male presented with a significant painless decreased vision in the right eye following an electrical injury of the right hand in his workplace. The best-corrected visual acuity (BCVA) of the right eye was 20/40. Funduscopic examination of the right eye revealed diffuse superficial and deep intraretinal hemorrhages, mild venous tortuosity, and an area of the pale retina. Optical coherence tomography (OCT) demonstrated hyperreflective band-like lesions in the middle retinal layers. Patchy areas of vascular flow void in deep capillary plexus seen in OCT angiography of the right eye were compatible with PAMM. Fluorescein angiography of the right eye was indicative of delayed venous filling suggestive of CRVO. The left eye was completely normal on exam and imaging.

**Conclusion:**

This report illustrates the occurrence of CRVO associated with PAMM following electric shock injury. Electrical injury leads to a wide range of retinal manifestations. Clinicians need to pay attention to any hyperreflectivity and thinning of middle retinal layers in OCT in cases with the presentation of sudden visual loss following electrical injuries.

## 1. Introduction

Electrical injuries lead to a wide range of ocular manifestations, from the eyelid, conjunctival, corneal, and lens involvement to posterior segment manifestations. Complications such as vitreous hemorrhage, retinal detachment, retinal edema, chorioretinal rupture, macular hole, Purtscher-like retinopathy and retinal vascular occlusive diseases are examples of retinal pathologies reported following electrical injuries [1–3].

The severity of the electrical damage depends on the electric current intensity, the exposure time, and the tissue resistance to the electricity [4]. The optic nerve and retina are low resistance tissues and therefore easily damaged by electrical current [5].

Several mechanisms have been proposed to explain how tissue damage occurs following electrical injuries. These mechanisms include direct transmission of the current, heat effect, tissue ischemia, and mechanical injury from electrical shock waves [6]. The electric current passes through the retinal pigment epithelium (RPE) cells, then generates heat and causes tissue damage; the absorption of the energy by the RPE cells can cause damage to the overlying retina. Because of a higher concentration of melanin in the macula (RPE cells are thicker and more compact), it causes more heat production and thermal damage. In addition, although the macula is the most affected area, the entire retina may be affected [7]. Here, we describe a case of unilateral PAMM and nonischemic CRVO following electrical injury.

## 2. Case Description

A 45-year-old male, an electrical technician by profession, presented to us with marked painless decreased vision in the right eye (RE) since the last day following an electrical injury in his workplace. A current of 400 V passed through his right hand before the presentation. There was no history of unconsciousness or falling after injury. No significant family or past medical history was documented. His general physical examination was normal.

On ocular examination, his best-corrected visual acuity (BCVA) was 20/40 for the right eye (RE) and 20/20 for the left eye (LE). The pupil examination revealed round and reactive pupils and a negative relative afferent pupillary defect.

Extraocular movements were normal. Intraocular pressure measured 12 mmHg for the RE and 13 mmHg for the LE. Anterior segment examination was unremarkable. We found diffuse superficial and deep intraretinal hemorrhages and mild venous tortuosity compatible with CRVO and an area of the pale retina in the macula of the RE. The examination of the LE was entirely normal.

**Figure 1:**
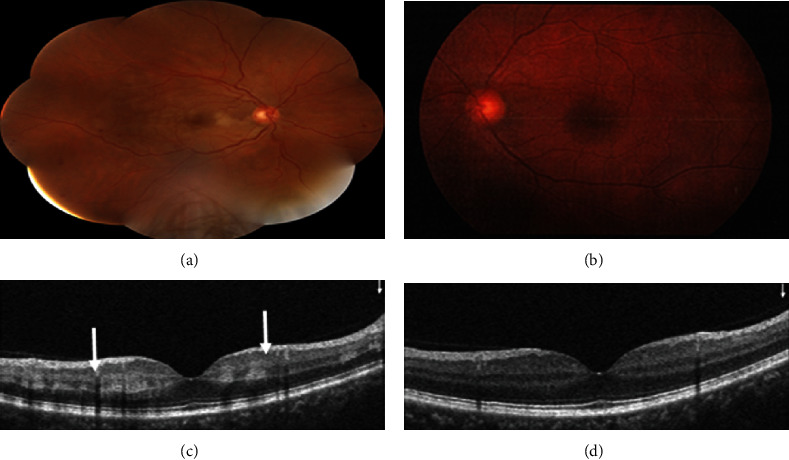
Color fundus photography (CFP) and spectral-domain optical coherence tomography (SD-OCT) of the right eye (a, c) and the left eye (b, d) at presentation. CFP of the RE (a) shows mild venous engorgement and widespread peripheral retinal hemorrhages located in all 4 quadrants and multiple small areas of pale retina in the macula. CFP of the left eye LE (b) does not show any retinal abnormalities. SD-OCT scan of the macula of the RE (c) shows hyperreflective band-like lesions in the middle retinal layers of the macula (arrows) of the RE and no abnormalities at the macula of the LE (d).

**Figure 2:**
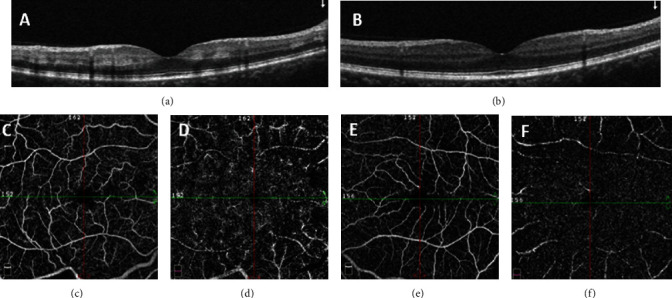
OCT angiography (OCT-A) with a scanning area of 6∗6 mm at presentation. OCT-A shows mild attenuation of the vascular flow signal in the superficial capillary plexus (SCP) (c) and patchy areas of vascular flow void in the deep capillary plexus (DCP) (d) of the RE and normal flow in the SCP (e) and DCP (f) of the LE.

**Figure 3:**
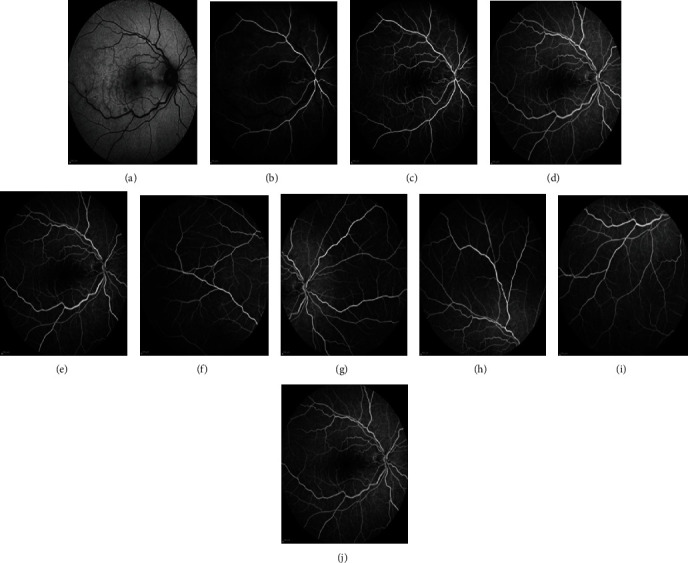
Fluorescein angiography frames of the right eye at presentation show delayed venous filling with the absence of macular edema or nonperfusion in the late phase of the angiogram.

Multimodal imaging including spectral-domain optical coherence tomography (SD-OCT) (Heidelberg Eye Explorer version 1.9.13.0, Spectralis Viewing Module 6.5.2.0; Heidelberg Engineering), OCT angiography (Optovue, Inc., Fremont, CA, USA, software version: 2018,0,0,18), and fluorescein angiography (Heidelberg Eye Explorer version 1.9.13.0, Spectralis Viewing Module 6.5.2.0; Heidelberg Engineering) has been performed to make a more accurate diagnosis. SD-OCT of the RE showed no macular edema and hyperreflective band-like lesions in the middle retinal layers suggestive of paracentral acute middle maculopathy (PAMM). (Figure 1). OCT angiography (OCT-A) with a scanning area of 6∗6 mm of the RE was indicative of attenuation of vascular flow signal in the superficial capillary plexus and patchy areas of vascular flow void in the deep capillary plexus (Figure 2). In fluorescein angiography (FA) of the RE, delayed venous filling was observed (Figure 3). No abnormality was detected in the imaging of the LE.

Our patient's BCVA was 20/20 in both eyes after a month of follow-up, and no neovascularization of the retina or iris had developed.

## 3. Discussion

PAMM was first described in 2013 by Sarraf et al. as a hyperreflective band-shaped lesion in the inner nuclear layer (INL) and the outer plexiform layer (OPL), leading to thinning of the INL. PAMM first was considered as a variant of acute macular neuroretinopathy (AMN), but these are now known as two distinct entities. Unlike PAMM, AMN lesions are red wedge-shaped intraretinal lesions that point toward the fovea and are better seen with infrared reflectance images. On the other hand, AMN lesions in OCT are placed deeper than PAMM and involve the outer nuclear layers and the OPL and may lead to thinning and destruction of the outer retinal layers. Although the exact mechanism of PAMM is still under investigation, OCT-A findings have suggested vascular changes in the intermediate and deep capillary plexus due to hypoperfusion. The INL and OPL are located in the watershed area of the retinal blood supply. In a hypoperfusion state, the maximum blood flow is directed toward the superficial capillary plexus and choroid, so INL and OPL are more prone to ischemia [8]. Also, the high oxygen demand of the horizontal cells in the middle retina made this area more sensitive to ischemic damage [9].

Another mechanism that is responsible for INL damage in PAMM is reperfusion injury. Reperfusion occurred following a transient hypoperfusion results in increased production of reactive oxygen species, nitric oxide neurotoxicity, inflammation, and extravascular leakage. Reperfusion injury is associated with chronic changes of PAMM [10].

Previous studies have shown the association between some retinal vascular diseases and PAMM including retinal artery and venous occlusion [11].

Electric current can damage tissue in several ways. The direct passage of electricity through the tissues leads to the destruction of the cell membrane and direct cell damage. Also, the heat produced by the conversion of electrical energy causes vascular contraction, resulting in tissue ischemia and eventually necrosis. Also, the direct mechanical injury caused by an electric shock wave is noted. The tissue conductivity of electrical current also affects the severity of the damage, so tissues such as the retina and optic nerve are more vulnerable to electrical injury. The macular region is more prone to electrical injury due to several reasons. The avascularity of the fovea makes it more sensitive to ischemic damage. Due to the greater accumulation of melanin in the macula, the heat produced in the macular region is higher, and the risk of tissue ischemia is more [4, 5].

It seems that in our patient, the transmission of electric current led to vascular constriction followed by tissue ischemia and presented with central retinal vein occlusion. Findings of the fundoscopic examination including diffuse superficial and deep intraretinal hemorrhages and mild venous engorgement and delayed venous filling in FA support the diagnosis. According to the abovementioned mechanisms, both hypoperfusion and reperfusion injury may disturb vascular flow toward the deep capillary plexus (DCP) and cause PAMM in this patient. Although FA has been the gold standard for the evaluation of retinal blood circulation, it is not able to show blood flow in the vascular layers of the retina separately. OCT-A is a noninvasive modality, which is helpful for this purpose. In our patient, hyperreflective, band-like lesions in the middle retinal layers of the macula in SD-OCT, and patchy areas of vascular flow void in the DCP in OCT-A are suggestive of PAMM.

There is yet to be a treatment that has been proved to reverse PAMM; thus, treatment should be focused on the underlying cause of PAMM when it is present [11]. In our case, CRVO was present as mentioned above. Intravitreal injections of antivascular endothelial growth factor (VEGF) and steroid implants are currently available treatments for macular edema secondary to CRVO. Patients with lower baseline VA (count finger or less) showed higher absolute improvement after treatment. Anti-VEGF injections, according to prior studies, are more preferable due to higher and more lasting visual outcome improvements as well as fewer side effects. Steroids would generally not be favored in individuals with an increased risk of glaucoma or younger phakic patients. On the other hand, more endophthalmitis rate is reported after intravitreal steroids compared to anti-VEGF injections [12]. Because there was no sign of macular edema in the physical examination or retinal imaging in the reported case and his baseline visual acuity was not particularly low, we decided on clinical follow-up without intervention.

As in our patient, the majority of PAMM cases have a good visual prognosis, with good final VA and no known neovascularization or rubeosis iridis in concomitant CRVO and CRAO cases [8].

## 4. Conclusion

The present case illustrates a patient with a sudden unilateral marked decrease in visual acuity following an electrical injury at the workplace. Findings of ocular examination and multimodal imaging support the diagnosis of PAMM and nonischemic CRVO. Electrical injuries lead to a wide range of retinal presentations that can be nonspecific. Ophthalmologists need to be familiar with the various ocular manifestations of electrical injuries. Paying attention to a history of electrical injury in an otherwise healthy person presented with PAMM may be helpful. This is the first report of PAMM following electric shock injury. Currently, this report does not prove any definite relationship between electrical injury and PAMM, and further studies are required for preparing a causal relationship.

## Data Availability

The retinal imaging data used to support the findings of this study are included within the article.
